# Bivalve-specific gene expansion in the pearl oyster genome: implications of adaptation to a sessile lifestyle

**DOI:** 10.1186/s40851-016-0039-2

**Published:** 2016-02-18

**Authors:** Takeshi Takeuchi, Ryo Koyanagi, Fuki Gyoja, Miyuki Kanda, Kanako Hisata, Manabu Fujie, Hiroki Goto, Shinichi Yamasaki, Kiyohito Nagai, Yoshiaki Morino, Hiroshi Miyamoto, Kazuyoshi Endo, Hirotoshi Endo, Hiromichi Nagasawa, Shigeharu Kinoshita, Shuichi Asakawa, Shugo Watabe, Noriyuki Satoh, Takeshi Kawashima

**Affiliations:** Marine Genomics Unit, Okinawa Institute of Science and Technology Graduate University, Onna, Okinawa 904-0495 Japan; DNA Sequencing Section, Okinawa Institute of Science and Technology Graduate University, Onna, Okinawa 904-0495 Japan; Pearl Research Institute, Mikimoto CO. LTD, Shima, Mie 517-0403 Japan; Graduate School of Life and Environmental Science, University of Tsukuba, Ibaraki, 305-8572 Japan; Department of Genetic Engineering, Faculty of Biology-Oriented Science and Technology, Kinki University, 930 Nishimitani, Kinokawa, Wakayama 649-6493 Japan; Department of Earth and Planetary Science, Graduate School of Science, The University of Tokyo, Bunkyo-ku, Tokyo 113-0033 Japan; Atmosphere and Ocean Research Institute, The University of Tokyo, Kashiwa, Chiba 277-8564 Japan; Department of Applied Biological Chemistry, Graduate School of Agricultural and Life Sciences, The University of Tokyo, Bunkyo-ku, Tokyo 113-8657 Japan; College of Life Sciences, Zhejiang University, Hangzhou, Zhejiang 310058 People’s Republic of China; Department of Aquatic Bioscience, Graduate School of Agricultural and Life Sciences, The University of Tokyo, Bunkyo-ku, Tokyo 113-8657 Japan; Kitasato University School of Marine Bioscience, Sagamihara, Kanagawa 252-0373 Japan; Present Address: Graduate School of Life and Environmental Science, University of Tsukuba, Ibaraki, 305-8572 Japan

**Keywords:** Pearl oyster, *Pinctada fucata*, Genome, Hox, ParaHox, Heat shock proteins, C1q, Biomineralization

## Abstract

**Introduction:**

Bivalve molluscs have flourished in marine environments, and many species constitute important aquatic resources. Recently, whole genome sequences from two bivalves, the pearl oyster, *Pinctada fucata,* and the Pacific oyster, *Crassostrea gigas*, have been decoded, making it possible to compare genomic sequences among molluscs, and to explore general and lineage-specific genetic features and trends in bivalves. In order to improve the quality of sequence data for these purposes, we have updated the entire *P. fucata* genome assembly.

**Results:**

We present a new genome assembly of the pearl oyster, *Pinctada fucata* (version 2.0). To update the assembly, we conducted additional sequencing, obtaining accumulated sequence data amounting to 193× the *P. fucata* genome. Sequence redundancy in contigs that was caused by heterozygosity was removed *in silico*, which significantly improved subsequent scaffolding. Gene model version 2.0 was generated with the aid of manual gene annotations supplied by the *P. fucata* research community. Comparison of mollusc and other bilaterian genomes shows that gene arrangements of Hox, ParaHox, and Wnt clusters in the *P. fucata* genome are similar to those of other molluscs. Like the Pacific oyster, *P. fucata* possesses many genes involved in environmental responses and in immune defense. Phylogenetic analyses of heat shock protein70 and C1q domain-containing protein families indicate that extensive expansion of genes occurred independently in each lineage. Several gene duplication events prior to the split between the pearl oyster and the Pacific oyster are also evident. In addition, a number of tandem duplications of genes that encode shell matrix proteins are also well characterized in the *P. fucata* genome.

**Conclusions:**

Both the *Pinctada* and *Crassostrea* lineages have expanded specific gene families in a lineage-specific manner. Frequent duplication of genes responsible for shell formation in the *P. fucata* genome explains the diversity of mollusc shell structures. These duplications reveal dynamic genome evolution to forge the complex physiology that enables bivalves to employ a sessile lifestyle in the intertidal zone.

**Electronic supplementary material:**

The online version of this article (doi:10.1186/s40851-016-0039-2) contains supplementary material, which is available to authorized users.

## Introduction

Bivalves are the second largest group in the Phylum Mollusca, outnumbered only by gastropods, and represent one of the most common animal groups in both marine and freshwater ecosystems. Notably, some bivalve species are abundant in littoral and shallow water environments, where they experience different types of fluctuating stresses, such as temperature, salinity, and oxygen concentration. Most adult bivalves employ a sessile, suspension-feeding life style, the energetic cost of which is lower than that of browsing [1]. Due to their aquatic habitats and filter feeding, they must defend themselves against microbial invasion by means of innate immune systems. In addition, sedentary bivalves cannot escape predation; therefore, they protect themselves with calcareous shells. These biological features of the bivalve adaptation strategy have resulted from expansion of specific gene families related to environmental response [2], immune defense [2–4], and biomineralization [5]. These findings raise the question of whether these gene expansions occurred in bivalve common ancestor or independently in various lineages.

The Genus *Pinctada* includes the pearl oysters, such as *Pinctada fucata*, *P. margaritifera*, and *P. maxima*, which are distributed in subtropical and tropical portions of the Indo-Pacific Ocean [6]. These species have been commercially farmed for pearl production since Kokichi Mikimoto established the pearl culture industry at the end of 19th century, using *P. fucata* [7]. Recently, transcriptomics [8, 9], proteomics [10–12], and gene knockdown techniques [13–15] have been used to investigate genetic components of shell and pearl biomineralization. Thus, mechanisms of pearl formation in *Pinctada* have been actively investigated for their economic potential, as well as their fascinating biology. Now, pearl oysters are becoming experimental model molluscs for biomineralization research.

In 2012, we decoded the draft genome of *Pinctada fucata* [16], one of the most important species for cultured pearl production in Asia. The genome of *P. fucata* has been thoroughly mined to find genes responsible for biomineralization [5], physiology [17], and reproduction [18]. A broad range of transcription factors [19–21] and signaling molecules [22] has also been investigated. These provide valuable information about lophotrochozoans to better understand evolution of Bilaterian body plans. Soon after publication of the *P. fucata* genome, genomes of the Pacific oyster, *Crassostrea gigas* [2] and the limpet, *Lottia gigantea* [23] were also published. The growing body of molluscan genome data provides an opportunity to characterize general and unique features among molluscs, for which sequence information has, until recently, been scant.

The present study produced a new version of the *P. fucata* genome assembly (version 2.0), which provides longer contigs and scaffolds, and more consecutive gene arrays compared to the previous version. To improve the assembly, additional sequence data were generated, and an advanced assembly strategy addressed the heterozygotic nature of the genome. Along with the establishment of a new genome assembly, we also generated gene model version 2.0. Information on gene annotation done manually by the *P. fucata* research community [24] was used for the gene model prediction.

In this report, we surveyed bivalve-specific genomic changes. We performed molecular phylogenetic analyses for gene families that have been expanded in bivalves, including heat shock protein 70 (HSP70) and C1q domain-containing proteins (C1qDC). In addition, we thoroughly investigated shell matrix protein (SMP) gene clusters, which were partly described in the previous version of the genome assembly [5]. We also verified conserved gene clusters for *Hox*, *ParaHox,* and *Wnt* genes among bilaterians using the new *Pinctada* genome assembly.

## Methods

### Genome sequencing and assembly

Genomic DNA, which is identical to that obtained in the previous study [16], was prepared for paired-end libraries and sequenced with an Illumina MiSeq and a Genome Analyzer IIx (GAIIx) [25]. Raw reads were quality trimmed using Trimmomatic 0.30 [26]. The whole-genome shotgun (WGS) and paired-end reads sequenced by Takeuchi et al. (2012) [16] and this study were assembled using GS De Novo Assembler, version 2.6 (Newbler, Roche) [27]. After removing redundant sequences from the contig assembly, paired-end and mate-pair sequences were added for scaffolding performed with SSPACE 1.1 [28]. Gaps in scaffolds were filled using GapCloser 1.12 [29]. See Additional file 1: note for more detail.

### Transcriptome sequencing and assembly

Transcriptome sequencing used in this study is described in Takeuchi et al. (2012) [16]. Additionally, a cDNA library of early developmental stages and adult tissues, including mantle, was prepared and sequenced with an Illumina GAIIx. All sequences were cleaned and trimmed with Trimmomatic 0.30 [26], and then assembled using Trinity (version r20140413p1) [30].

### Gene prediction, annotation, and identification of gene families

The resulting genome assembly (*P. fucata* genome ver. 2.0) and transcriptomic data were used for *de novo* gene model prediction with PASA (version r20130907) [31] and AUGUSTUS 3.0.2 [32] platforms, as described previously [16]. Gene annotation information manually confirmed in Pearl Oyster Annotation Jamborees [24] was added in order to train the gene prediction algorithm and to generate a hint file for AUGUSTUS. Gene models that encoded more than 49 amino acids were retained. Sequences significantly similar to transposable elements were detected with CENSOR 4.2.28 [33] and excluded from the gene model. Gene models of *P. fucata*, *Lottia gigantea* [23], and *Crassostrea gigas* [2] were assigned to the ortholog group of the OrthoMCL Database version 5 [34, 35]. Three molluscan gene models that were not assigned to the OrthoMCL ortholog group were then clustered with local OrthoMCL software in order to identify mollusc-specific gene families. Next, gene models that did not cluster with others (“orphan gene models”) were examined with BLASTN against *P. fucata* transcriptomic sequences. Orphan gene models without transcriptomic evidence were excluded from the final gene model set, named gene model, version 2.0.

Gene model version 2.0 was BLASTN-searched against version 1.0 models and the best match model was regarded as synonymous. Hox, ParaHox, Wnt, and SMP genes were annotated by referring to previous studies [5, 21, 22]. SMP genes were also searched with BLASTP against SMPs of *P. margaritifera* and *P. maxima* [10] and were annotated manually. BLASTP searches against the UniProtKB and NCBI non-redundant (nr) databases were also conducted in order to confirm annotations. Results of BLASTP searches (E-value threshold 1e^-5^) against protein sequences of the UniProtKB database, were analyzed with PANTHER [36] for GO annotation. GO enrichment of genes conserved between *P. fucata* and *C. gigas*, but not *L. gigantea*, was investigated by calculating a *P* value based on hypergeometric distribution.

### Molecular phylogeny of expanded gene families

Conserved domains of proteins predicted in the *Pinctada fucata* genome were searched against the Pfam database (Pfam-A.hmm, release 24.0; http://pfam.xfam.org/) using HMMER 3.0 [37]. Representative animal genomes were also surveyed for comparison and phylogenetic analysis. The following protein sequences from public databases were retrieved for this study; *Acropora digitifera* (http://marinegenomics.oist.jp/), *Lottia gigantea*, *Helobdella robusta*, *Capitella teleta*, *Daphnia pulex*, and *Nematostella vectensis* (http://genome.jgi.doe.gov/), *Crassostrea gigas* (http://gigadb.org), *Hydra magnipapillata*, *Amphimedon queenslandica* (http://www.ncbi.nlm.nih.gov) and *Caenorhabditis elegans*, *Drosophila melanogaster*, *Branchiostoma floridae*, *Strongylocentrotus purpuratus*, and *Homo sapiens* (http://www.genome.jp). Amino acid sequences of *Mytilus galloprovincialis* C1q domain-containing proteins were downloaded from NCBI. Amino acid sequences were aligned with Muscle [38], then maximum likelihood trees were constructed with RAxML 8.1.3 [39]. The best-fit model of amino acid evolution for each tree was selected using ProteinModelSelection.pl script provided in the RAxML package. One hundred bootstrap replicates were generated.

## Results and discussion

### New genome assembly

To update the assembly, additional sequencing was conducted. Results of genome sequencing are summarized in Additional file 1: Table S1. High-throughput sequencing added more than 1 billion reads, and accumulated sequence data represented ~193× the *P. fucata* genome, which has an estimated genome size of 1.14Gb [16]. The *P. fucata* genome is known to be highly heterozygotic, which complicates assembly [16]. To address this, we removed redundant contigs with low sequence coverage depth before scaffold construction (see Additional file 2: Figure S1 and Additional file 3 for detail). As a result, the final genome assembly (version 2.0) achieved contig and scaffold N50 sizes of 21.3 kb and 167 kb, which are respectively 20× and 11× longer than those of the previous version (version 1.0), (Table 1 and Additional file 1: Table S2). Our results show that contig filtering based on sequence similarity and coverage depth is a very efficient way to reduce redundancy of the assembly and to improve subsequent scaffolding. In consequence of the scaffold elongation, gene clusters and tandem arrangement of genes were clarified, as discussed below.

**Table 1:** Summary of the *P. fucata* genome assembly version 2.0

Contigs	
Number of contigs	77192
Total length of sequences	760 Mb
Average contig length	9.8 kb
Contig length N50	21.3 kb
Scaffolds	
Number of scaffolds	29306
Total length of sequences	815 Mb
Total gap length	55 Mb
Average scaffold length	27.8 kb
Scaffold length N50	167.0 kb

### New gene models

In the new *P. fucata* genome assembly, 30,619 gene models were initially predicted using AUGUSTUS software. Clustering with OrthoMCL shows that 5,096 gene models are not grouped with any other genes in OrthoMCL DB, in the *Lottia*, *Crassostrea*, and *Pinctada* genomes. Among these “orphan” gene models, sequences of 1,266 models were not found in the merged transcriptomic assemblies. We regarded them as faultily predicted gene models and filtered them out of the final gene model. Accordingly, 29,353 models were retained and 23,516 (80.1 %) represent putative, full-length genes with both start and stop codons (Table 2). The average number of exons per gene was more than double that in the previous version. Among all gene models, 19,533 (66.5 %) were BLASTP hits to the UniProtKB database (e-value cutoff 1e^-5^).

**Table 2:** Comparison of gene model versions of *P. fucata*

	Ver. 1.0 Takeuchi et al. [16]	Ver. 2.0 This study
Number of gene models	43760	29353
Number of full length gene models	23257 (53.1 %)	23516 (80.1 %)
Number of exons per transcript	3.16	7.53
Average range per gene model (bp)	6960	12203

**Fig. 1:**
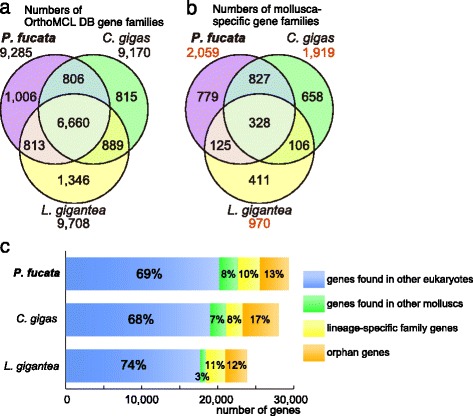
Lineage-specific genes/gene families are more numerous than conserved gene families among molluscs. **a** The number of common gene families assigned using OrthoMCL DB. **b** The number of gene families not assigned using OrthoMCL DB, but detected among mollusc species. **c** Gene composition of the three mollusc genomes. Bars represent the number of genes that are either members of common gene families (assigned to an OrthoMCL DB gene family; *blue*), shared families (not assigned using OrthoMCL DB, but shared by at least two mollusc species; *green*), lineage-specific families (present only in a single mollusc genome; *yellow*), and orphan genes (with no putative homolog; *orange*)

**Fig. 2:**
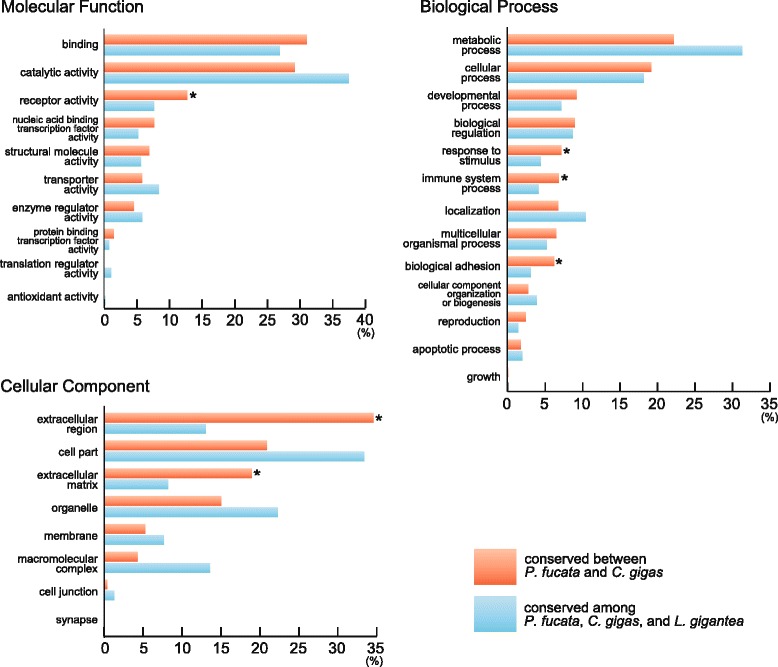
Enriched GO categories may reflect the sedentary lifestyles of bivalves in the intertidal zone. The percentage of all annotated genes is shown. Asterisks indicate GO categories that are significantly (*p* < 0.01) enriched in bivalve-specific gene models

The numbers of gene family classified by orthoMCL software are shown in Fig. 1a and b. The numbers of genes included in each gene family are presented in Additional file 2; Figure S2. The new *P. fucata* gene model contains 20,179 genes, assigned to 9,285 gene families using orthoMCL DB Version 5 (Fig. 1a). Of these, 6,660 gene families, which include 15,998 *P. fucata* genes, are shared among the three molluscs (Fig. 1a, Additional file 2: Figure S2a). While orthoMCL DB Version 5 includes as many as 150 organismal genomes, only one lophotrochozoan genome (*Schistosoma mansoni*) is present in the database. In order to identify mollusc-specific gene families, we collected and grouped the *Pinctada*, *Crassostrea*, and *Lottia* genes that were not assigned to an orthoMCL gene family (Fig. 1b). We analyzed the remaining 9,174 gene models (29,353–20,179) that were not assigned in orthoMCL DB, and assigned an additional 5,344 gene models into 2,059 novel gene families (Fig. 2b, Additional file 2: Figure S2d). Finally we categorized the residual 3,830 gene models (9,174–5,344) as orphan gene models since they do not have any sequence similarity to known genes (Fig. 1c). *Pinctada*-specific (779), *Crassostrea*-specific (658), and bivalve-specific (827) gene families were detected, while gene families shared among the three molluscs (328) were less numerous. This feature is more apparent when comparing the number of genes corresponding to each gene family group. In the case of *P. fucata*, only 437 genes belong to shared gene families, while 2,877 genes are unique to *P. fucata* (Additional file 2: Figure S2d). In other words, novel genes that emerged in the common ancestor of bivalves and gastropods are less numerous than genes generated by *Pinctada*-lineage-specific gene expansion. Only 3–8 % of genes in the molluscan genomes are mollusc-specific and shared by molluscs, while more than 20 % are lineage-specific genes (Fig. 1c). Thus, novel genes, acquired and duplicated at the class (Classes Bivalvia and Gastropoda) or lower phylogenetic level, characterize these molluscan genomes.

In order to predict functions of genes conserved between the two bivalves or among all three molluscs, GO annotation was conducted (Fig. 2). Genes that were related to “receptor activity,” “response to stimulus,” “immune system process,” and “extracellular region” were more abundant in bivalves compared to those of genes shared by all three molluscs. This suggests that several gene families related to environmental response and immune system are expanded in bivalves. Similarly, bivalve-specific gene expansion corresponding to “extracellular region” and “extracellular matrix” was evident, and these events were responsible, at least in part, for evolution of bivalve biomineralization-related genes. In addition, genes assigned to “biological adhesion” are significantly enriched in the bivalve lineage, possibly reflecting their sedentary lifestyles.

### Gene expansion of heat shock protein 70 and C1q domain-containing proteins

Recent genomic and transcriptomic surveys have shown that some gene families involved in responses to environmental change or microbial attack, are greatly expanded in bivalves, including *Crassostrea* [2] and *Mytilus* [3, 40, 41]. However, it has been unclear whether gene expansion events are lineage-specific or common to all bivalves.

**Fig. 3:**
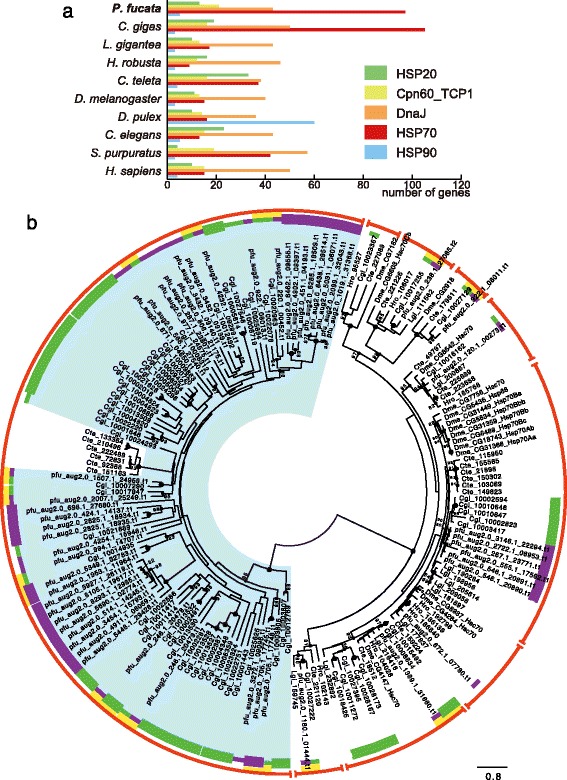
Expansion of heat shock protein 70 (HSP70) genes has occurred in bivalve genomes. **a** Genes that contain conserved Pfam domains related to heat shock chaperones. See also Additional file 1: Table S4. **b** Unrooted maximum likelihood molecular phylogeny of HSP70 domain sequences of selected animal genomes. The blue area highlights a group of predominantly bivalve genes. Protein sequences of *Pinctada fucata* and *Crassostrea gigas* are marked with purple and green lines, respectively. Thick purple and green lines indicate that the clade comprises only one bivalve species, suggesting lineage-specific gene expansion. Yellow lines designate gene pairs of *P. fucata* and *C. gigas* supported by high bootstrap values (≥80 %), indicating that the genes probably existed in the common ancestor of the two bivalves. Nine outer red arcs, except the largest one on the left, show groups composed of four or more protostome genes. Nodes of the tree supported with high bootstrap values (≥80 %) are marked with black dots with the number, while larger black dots without a number indicate 100 % bootstrap support. The scale bar represents expected substitutions per site in the aligned regions. The first three letters of the protein ID indicate the species name; pfu: *P. fucata*, Cgi: *C. gigas*, Lgi: *Lottia gigantea*, Hro: *Helobdella robusta*, Cte: *Capitella teleta*, and Dme: *Drosophila melanogaster*. HSP70 domain sequences with lengths of 200 amino acids or more were used for analysis with the WAG + GAMMA + F model

Heat shock proteins (HSPs) are molecular chaperones that maintain protein folding, and that rescue proteins that have been denatured or damaged by environmental or physiological stresses [42, 43]. The number of HSP70 genes is greatly increased in the oyster genome [2], which may enable them to survive in intertidal zones, where they are exposed to air and to significant temperature changes during tidal cycles. In fact, the HSP70 gene family is also expanded in the pearl oyster genome, while the numbers of genes with other heat shock chaperon domains such as HSP20, HSP90, and DnaJ are comparable to those of gastropods, annelids, and other animals (Fig. 3a, Additional file 1: Table S3).

We reconstructed a molecular phylogenetic tree of HSP70 proteins of five lophotrochozoan (three molluscs and two annelids) and fly genomes (Fig. 3b). The tree clearly shows two distinct groups: one is ancestral and the other is almost completely composed of bivalve genes (Fig. 3b). HSP70 genes of a polychaete, *Capitella teleta,* are also included in the latter group. Although the evolutionary relationship between these bivalve and polychaete genes is indeterminate because of the low bootstrap value, we speculate that the bivalve-dominant gene group was derived from the ancestral gene shared by molluscs and polychaetes. It then expanded in the bivalve lineage while being lost in the gastropod lineage. In the bivalve-dominant gene group, nine pairs of *P. fucata* and *C. gigas* genes are closely associated (Fig. 3b). This topology suggests that they are orthologous pairs and that these nine bivalve-specific HSP70 genes were present prior to the divergence of the *Pinctada* and *Crassostrea* lineages, which date back approximately 400 million years [44]. Furthermore, species-specific gene expansions are also observed (Fig. 3b). At least six gene families in the tree suggest independent gene expansion events in *P. fucata*, while eleven more suggest the same in *C. gigas*. Pearl oysters are mainly found in the subtidal zone, which is a more stable environment than the intertidal zone because it is always inundated. However, the subtidal and intertidal zones are still very stressful environments, and sessile bivalves (the pearl oyster and the Pacific oyster) need to cope with adverse conditions in shallow water, such as salinity and temperature changes caused by weather. Nutrient and oxygen concentrations are also changed by plankton blooms. Therefore, the independently expanded HSP70 gene family in both *P. fucata* and *C. gigas* may underlie adaptation to a sessile lifestyle in such fluctuating and stressful conditions. Moreover, aside from the bivalve-dominant group, eight groups that include HSP70 genes of four or more protostome species, are recognized (Fig. 3b), six of which are supported by high bootstrap values (≥80 %). Therefore, it is likely that the common ancestor of protostomes had approximately eight or nine HSP70 genes, and evolutionary radiation of this gene family has occurred in each lineage.

**Fig. 4:**
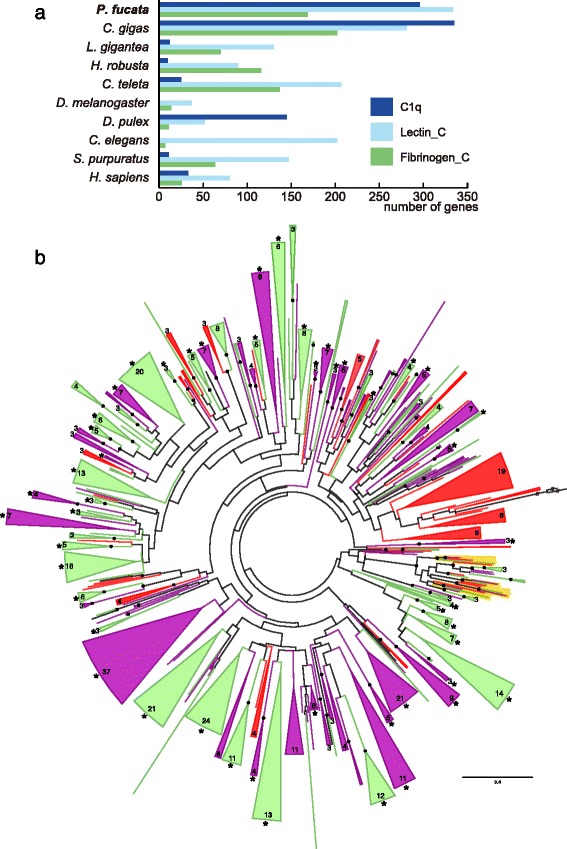
Tandem duplication and expansion of genes related to innate immune recognition has occurred in all three bivalve lineages. **a** Genes that contain selected Pfam domain candidates related to recognition of non-self antigens. See also Additional file 1: Table S7. **b** Unrooted maximum likelihood molecular phylogeny of C1q domain sequences of three mollusc genomes (*Pinctada fucata*: purple, *Crassostrea gigas*: green, and *Lottia gigantea*: black), and MgC1q proteins of *Mytilus galloprovincialis* (red). Wedges indicate the merged gene group, which is composed of sequences only from bivalve species, and the number of constituent genes shown in the arc. Wedges without numbers indicate groups that include two genes. Merged groups that contain two or more tandemly arranged genes in the genome are marked with asterisks. Branches showing orthologous gene groups of three bivalves are highlighted in yellow. Nodes supported with high bootstrap values (≥80 %) are marked with black dots. Branches with slashes were collapsed in order to fit the tree onto the page. The scale bar represents expected substitutions per site in the aligned regions. C1q domain sequences with lengths of 100 amino acids or more were used for analysis with the WAG + GAMMA + F model

In the innate immune system, a wide repertoire of proteins is thought to be responsible for detecting pathogens and eukaryotic parasites via direct contact with surface epitopes or pathogen-associated molecular patterns (PAMPs) [45]. For example, microbial recognition capability has been described in many molluscan proteins including C1q domain-containing (C1qDC) proteins [46–48], fibrinogen-related proteins (FREPs) [49, 50], and lectins [51–53]. Bivalve genomes possess a large number of candidate genes that encode functional domains related to recognition of PAMPs (Fig. 4a and Additional file 1: Table S4). In particular, the number of C1q genes is enormously expanded in the *P. fucata* genome, consistent with the *C. gigas* genome [4] and a mussel, *Mytilus galloprovincialis,* transcriptome [3]. A Pfam domain-search detected 296 gene models with C1q domains in the *P. fucata* genome, and 335 in that of *C. gigas*, while only 12 such models were found in the *L. gigantea* genome. Multiple, lineage-specific gene expansions of C1q domain sequences are detected with high bootstrap values in the two bivalve genomes and the transcriptome of *M. galloprovincialis* [3] (Fig. 4b). Moreover, many C1qDC gene-pairs are tandemly arranged in the same scaffolds (Fig. 4b), suggesting that gene duplication events have occurred frequently in bivalve genomes. While the overall tree is supported by a low bootstrap value due to the short sequences (fewer than 120 amino acids) of the C1q domain, three sets of orthologous genes among bivalve species are detected (Fig. 4b), suggesting that these C1qDC genes became duplicated in the common ancestor of the three bivalves after their divergence from the gastropod lineage.

These phylogenetic analyses demonstrate that gene duplication events occurred prior to the divergence of the bivalve species examined here, and that lineage-specific gene expansion is also common in bivalve genomes. This flexible expansion of gene families has generated an immense gene repertoire associated with stress responses and immune defense. By contrast, the limpet, *Lottia*, did not acquire enlarged gene families, despite its similar habitat in the littoral zone. We suggest that because bivalves cannot escape from the adverse conditions, they must respond to the variable environment by having expanded HSP70 gene family. Their suspension-feeding system is protected from wide range of invading microorganisms. Gene expansion allows bivalves to settle in dynamic marine environments, such as the intertidal and subtidal zones.

### Tandem duplication of genes responsible for shell formation

Shell formation is one of the unique features of molluscs. The process is highly controlled by the organism by secretion of an organic shell matrix, which generates an organic framework that regulates calcification of the shell [54, 55]. Shell matrix proteins (SMPs) are considered the major components of the organic shell matrix, and SMP evolution is implicated in diversification of mollusc shell characters, including morphology, microstructure, and crystal polymorphism [10, 56].

**Fig. 5:**
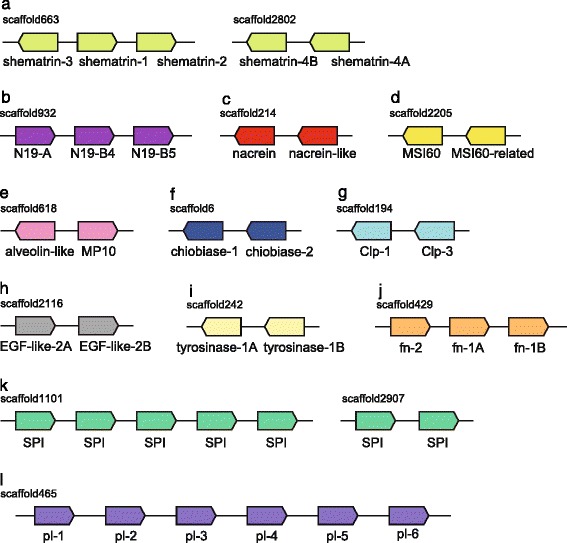
SMP gene families are clustered in the *P. fucata* genome. Relative position and orientations of genes are indicated. Lengths of scaffolds, genes, and intergenic regions are not to scale. **a** Shematrin. **b** N19. **c** Nacrein and nacrein-like. **d** MSI60 and MSI60-related. **e** Alveolin-like and MP10. **f** Chiobiase. **g** Chitinase-like. **h** EGF-like (**i**) Tyrosinase. **j** Fibronectin domain-containing. **k** Serine protease inhibitor. **l** Peroxidase-like. See also Additional file 1: Table S5 for detail

Using the previous sequence data from *P. fucata*, Miyamoto et al. (2013) generated a comprehensive list of shell formation-related genes, and showed that some SMP genes are tandemly arranged in the scaffold [5]. We searched further for SMP gene clusters in the new genome assembly and found an additional 14 duplicated loci for 12 SMP gene families (Fig. 5 and Additional file 1: Table S5). Of these genes, five encoding the Shematrin family, which contains repetitive and glycine-rich proteins [57], are tandemly arranged in two scaffolds (Fig. 5a). Three genes encoding N19 [58] are also clustered in a scaffold (Fig. 5b). Genes encoding Nacrein-like and MSI60-related [5, 59, 60] proteins are detected with BLAST searches, and they are located adjacent to their relatives (Fig. 5c and d). So far, there is no direct evidence that Nacrein-like and MSI60-related proteins are involved in shell formation, and further functional analysis of these proteins is needed.

Orthologous genes that encode SMPs, reported from *P. margaritifera* and *P. maxima* [10], were also investigated. Interestingly, a number of SMP genes are tandemly arranged in the scaffolds (Fig. 5 and Additional file 1: Table S5). Pairs of genes encoding alveolin-like and MP10, chiobiase, chitinase-like protein (Clp), EGF domain-containing protein (EGF-like), and tyrosinase are present (Fig. 5e-i). Likewise, more than two tandem gene clusters of fibronectin domain-containing protein (fn), Kunitz/BPTI serine protease inhibitors (SPI), and peroxidase-like (pl) proteins are identified (Fig. 5j-l). These results show that SMP genes were frequently duplicated in the *Pinctada* genome. Tandemly arranged genes that encode SMPs (EGF domain-containing protein, peroxidase, and uncharacterized proteins) are also evident in the *Lottia* genome [61], indicating that tandem duplication of SMP genes is a common feature of bivalve and gastropods. Although the precise function of these genes in shell formation remains unknown, duplication and rapid molecular evolution [9, 62] of SMP genes may be a key feature for understanding the diversification of mollusc shell structure. It is also possible, however, that molecular functions of duplicated SMPs are redundant, and that increased gene copy number results in larger numbers of transcripts [62], to accelerate shell formation. Indeed, we reported an example in which tandem arrays of SMP genes were expressed in coordinated fashion [63]. In order to resolve the complexity of molecular evolution and functional diversification of SMPs, proteomic analysis of the *P. fucata* shell and a genome-wide gene expression survey of mantle are underway.

Among the SMP gene families discussed above, genes homologous to MSI60, Shematrin, and N19 are absent in the *C. gigas* genome. Likewise, another tandemly duplicated gene family, N16 [5], is not found in the oyster genome. In other words, these SMP gene families are unique to the *P. fucata* lineage. As mentioned before, abundant lineage-specific gene families are a feature of molluscan genomes (Fig. 1b). These gene families emerged and became duplicated in *P. fucata* lineage after the split of the pearl oyster and Pacific oyster lineages. Alternatively, it is possible that these two bivalves share an ancestral SMP gene, and that the gene evolved so rapidly that, at present, SMP genes in two bivalve genomes are significantly different from each other. In either case, rapid molecular evolution and a diverse repertoire of SMPs made possible the great variety of molluscan shell structures.

### Conserved clusters of Hox, ParaHox, and Wnt genes in the *P. fucata* genome

**Fig. 6:**
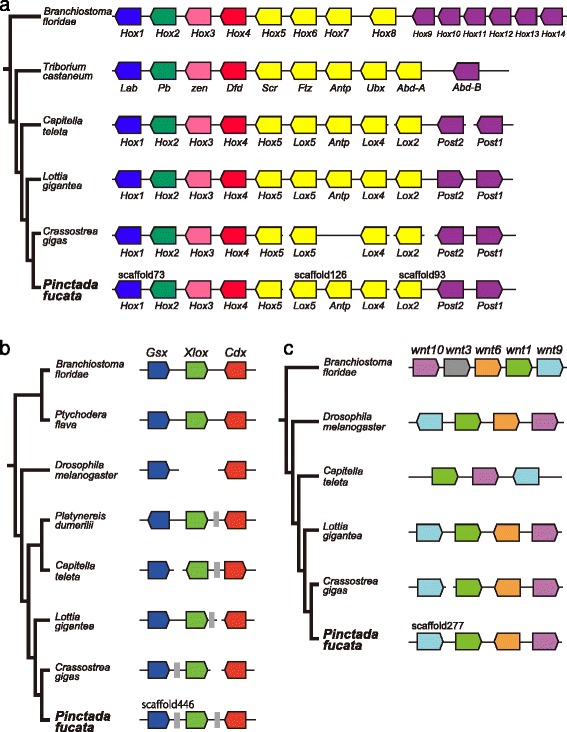
Hox, ParaHox, and Wnt gene clusters in the *P. fucata* genome resemble those of other protostomes. The relative position and orientation of the genes are indicated. **a** Hox gene cluster. *P. fucata* Hox genes are located on 3 scaffolds. **b** ParaHox gene cluster. *P. fucata* ParaHox genes are aligned on a single scaffold. The gray box indicates a non-ParaHox gene. **c** Wnt gene cluster. *P. fucata* Wnt1, 6, 9, and 10 genes are found on a single scaffold and the gene order is the same as that of *L. gigantea*. Lengths of scaffolds, genes, and intergenic regions are not to scale. See also Additional file 1: Tables S6-S8 for details

Hox, ParaHox, and Wnt gene clusters are the most conserved synteny blocks among bilaterian genomes [64–66]. To ascertain whether the pearl oyster genome preserves these gene clusters, the new genome assembly was thoroughly surveyed. Previously, gain and loss of Hox genes in molluscan classes were reported [67]. All 11 Hox gene transcripts were identified in *Pecten maximus* [68] while *Antp* was lost in the oyster genome [2]. In the *P. fucata* genome assembly, all 11 Hox genes are clustered in three scaffolds (Fig. 6a). The Hox gene, *LoxZ* [21], is located between *Lox5* and *Lox4*. We retrieved a longer sequence of the corresponding gene model from the new genome assembly, and a BLAST search confirmed that the gene actually encodes *Antp* (Additional file 1: Table S6). There are two non-Hox gene models upstream of *Hox5* in scaffold 73, and nine non-Hox gene models are present upstream of *Lox4* in scaffold 126 (Additional file 1: Table S6). As a result, the *P. fucata* Hox cluster is divided into three genomic regions. Two interruptions comprising non-Hox flanking genes between *Hox5* and *Lox5*, and between *Lox4* and *Lox2* are also observed in the *C. gigas* genome (Fig. 6a) [2], indicating that this feature of the Hox cluster occurred in the common ancestor of these two bivalves after their divergence from gastropods.

ParaHox genes, *Gsx*, *Xlox*, and *Cdx* are found in a single scaffold (Fig. 6b), which is the first indication of close linkage of the three ParaHox genes in molluscan genomes. These genes are separated by non-ParaHox gene models, which are also observed in the *Lottia* and *Crassostera* genomes (Fig. 6b and Additional file 1: Table S7). We also found a cluster of Wnt gene family genes (Fig. 6c and Additional file 1: Table S8). The tandem arrangement of *wnt9*, *1*, *6*, and *10* genes is identical to that of the limpet genome [69]; therefore this arrangement is considered the ancestral state in bivalves and gastropods. These results demonstrate that Hox, ParaHox, and Wnt gene clusters in the *P. fucata* genome are comparable to those of other molluscan genomes.

## Conclusions

We performed comparative genomic analyses using accessible molluscan genomes with our newly updated genome assembly of the pearl oyster, *Pinctada fucata*. Genes common to the two bivalves include a larger number of genes potentially relevant to extracellular matrix, environmental responses, and immune systems than are seen in a gastropod and other protostomes (Fig. 2). Consistently, protein-domain surveys and molecular phylogenetic analyses reveal extensive gene duplication of stress response genes (HSP70 in Fig. 3, C1qDC in Fig. 4). A survey of gene arrangements confirmed that frequent gene duplication of shell matrix proteins has occurred in bivalves (Fig. 5). All of these results suggest that adaptive changes in extant bivalve genomes have occurred in a species-specific manner. We also confirmed relatively conserved clusters of Hox, ParaHox, and Wnt genes among protostomes (Fig. 6). The revised pearl oyster genome provides insights into the molecular basis of oyster physiology and radiation.

### Availability of supporting data

All reads generated by this study have been deposited in the Sequence Read Archive (SRA) database (http://trace.ncbi.nlm.nih.gov/Traces/sra/) under the accession IDs DRP000496 and DRP000497 .

## Additional files

Additional file 1:
**Table S1.** Summary of *Pinctada fucata* genome sequence data. **Table S2.** Summary of the *Pinctada fucata* genome assemblies. **Table S3.** Numbers of genes containing functional domains related to heat shock proteins. See also Fig. 3a. **Table S4.** Numbers of genes containing functional domains related to non-self recognition and signaling. See also Fig. 4a. **Table S5.** List of biomineralization-related genes tandemly arranged in the genome. See also Fig. 5. **Table S6.** Hox and neighboring gene models tandemly arranged in the genome. **Table S7.** ParaHox and neighboring gene models tandemly arranged in the genome. **Table S8.** Wnt and adjacent gene models tandemly arranged in the genome. (PDF 385 kb) Additional file 2: 
**Figure S1.** Histograms of sequence coverage depth of each contig. (a) Contig coverage distribution in the initial assembly. The peak at lower coverage (near 26.5x) indicates redundant contigs caused by the heterozygotic nature of the *Pinctada fucata* genome. The red line shows the fitted normal distribution, where μ = 26.5 and σ = 3. (b) The histogram of contig coverage depth after redundant contigs were removed. (c) The histogram of scaffold coverage depth. These data indicate that redundant sequences in the genome assembly were effectively removed by a sequence similarity and coverage-based method. **Figure S2.** The numbers of genes corresponding to grouped gene families presented in Fig. 2a and b in the main text. (a–c) The numbers of genes that were assigned by OrthoMCL DB; (a) *Pinctada fucata*, (b) *Crassostrea gigas*, and (c) *Lottia gigantea*. (d–f) The numbers of genes that were not assigned by OrthoMCL DB, while detected in comparisons among three mollusc genomes; (d) *P. fucata*, (e) *C. gigas*, and (f) *L. gigantea*. Mollusc-specific genes shared by all three molluscs are much less abundant than lineage-specific genes. (PDF 213 kb) Additional file 3:
**Assembly strategy.** (PDF 97 kb)

## Abbreviations

C1qDC: C1q domain-containing protein
Clp: Chitinase-like protein
fn: fibronectin domain-containing protein
FREP: fibrinogen-related protein
HSP: heat shock protein
PAMP: pathogen-associated molecular pattern
pl: peroxidase-like
SMP: shell matrix protein
SPI: serine protease inhibitor
WGS: whole-genome shotgun
