# Supplementary material for: Bivalve-specific gene expansion in the pearl oyster genome: implications of adaptation to a sessile lifestyle
Source: Zoological Lett. 2016 Feb 18;2:3. doi: 10.1186/s40851-016-0039-2 (PMC4759782; doi:10.1186/s40851-016-0039-2)
Supplement: Additional file 1: — Table S1. Summary of Pinctada fucata genome sequence data. Table S2. Summary of the Pinctada fucata genome assemblies. Table S3. Numbers of genes containing functional domains related to heat shock proteins. See also Fig. 3a. Table S4. Numbers of genes containing functional domains related to non-self recognition and signaling. See also Fig. 4a. Table S5. List of biomineralization-related genes tandemly arranged in the genome. See also Fig. 5. Table S6. Hox and neighboring gene models tandemly arranged in the genome. Table S7. ParaHox and neighboring gene models tandemly arranged in the genome. Table S8. Wnt and adjacent gene models tandemly arranged in the genome. (PDF 385 kb) [file 40851_2016_39_MOESM1_ESM.pdf]

Table S1. Summary of *Pinctada fucata* genome sequence data.

Sequencer	Library	Total sequences (Gbp)	Number of reads (million)	Average read length (bp)	Estimated coverage depth
454	shotgun	15.2	44.5	341	16x
	4kb paired-end	1.1	3.8	286	
	10kb paired-end	2.4	7.8	309	
GAIIx	Shotgun	3.9	76	50	126x
	1kb paired-end	117.4	783	150	
	3kb mate-pair	10.9	216	50	
	10kb mate-pair	11.9	238	50	
Miseq	700pb paired-end	58.3	227.6	250	51x

New DNA libraries prepared for the present study are shaded in gray.

Table S2. Summary of the *P. fucata* genome assemblies.

Version	ver 1.0 (Takeuchi *et al*. 2012)[17]	**ver. 2.0 (This study)**			-	-
Software	Newbler + SSPACE	Newbler + SSPACE + GapCloser			SOAPdenovo	Platanus
		before redundancy removal of contigs	after redundancy removal & scaffolding	after gap closure		
**Contigs**						
Number of all contigs	1085563	512705	183966	77192	5422904	6903171
Number of short contigs (< 1kb)	765607	194080	27925	7931	5196356	6600868
Number of long contigs (> 10kb)	1223	3556	12828	23075	44	688
Total length of sequences (Mb)	1024Mb	988Mb	712Mb	760Mb	1551Mb	1811Mb
Contig length N50 (kb)	1.6kb	2.7kb	6.0kb	21.3kb	391bp	422bp
**Scaffolds**						
Number of scaffolds	800982	-	29306	29306	3971774	4413364
Number of short scaffolds (< 1kb)	657943	-	6115	6115	3578328	4277817
Number of long scaffolds (> 100kb)	629	-	2388	2377	14809	1401
Total length of sequences (Mb)	1413Mb	-	817Mb	815Mb	12492Mb	1589Mb
Total gap length (Mb)	387Mb	-	106Mb	55Mb	11032Mb	108330911
Scaffold length N50 (kb)	14.5kb	-	167.4kb	167.0kb	47.3kb	1.3kb
Longest (bp)	698791bp	-	1168043bp	1158730bp	434282bp	637412bp

Table S3. Numbers of genes containing functional domains related to heat shock proteins. See also Figure 3a.

Pfam accession	Pfam family identifier	***Pinctada fucata***	*Crassostrea gigas*	*Lottia gigantea*	*Helobdella robusta*	*Capitella teleta*	*Drosophila melanogaster*	*Daphnia pulex*	*Caenorhabditis elegans*	*Strongylocentrotus purpuratus*	*Branchiostoma floridae*	*Homo sapiens*	*Hydra magnipapillata*	*Nematostella vectensis*	*Acropora digitifera*	*Amphimedon queenslandica*
PF00011	HSP20	13	19	10	16	33	11	10	23	4	7	10	16	20	3	0
PF00118	Cpn60_TCP1	21	16	13	12	16	13	14	15	19	13	15	14	21	17	16
PF00226	DnaJ	43	50	43	46	38	40	36	43	57	42	50	41	48	48	55
PF00012	HSP70	97	105	17	9	37	15	16	13	42	19	15	11	13	23	87
PF00183	HSP90	5	3	3	3	4	3	60	5	3	3	4	5	4	4	3

Table S4. Numbers of genes containing functional domains related to non-self recognition and signaling. See also Figure 4a.

Pfam accession	Pfam family identifier	***Pinctada fucata***	*Crassostrea gigas*	*Lottia gigantea*	*Helobdella robusta*	*Capitella teleta*	*Drosophila melanogaster*	*Daphnia pulex*	*Caenorhabditis elegans*	*Strongylocentrotus purpuratus*	*Branchiostoma floridae*	*Homo sapiens*	*Hydra magnipapillata*	*Nematostella vectensis*	*Acropora digitifera*	*Amphimedon queenslandica*
PF00386	C1q	296	335	12	10	25	1	145	0	11	42	33	4	3	5	0
PF00147	Fibrinogen_C	169	202	70	116	137	14	11	7	64	214	26	22	128	109	211
PF00059	Lectin_C	334	281	130	90	207	37	52	202	147	645	80	73	64	99	1
PF01582	TIR	125	96	71	13	84	10	8	2	243	85	23	6	14	36	6
PF00531	Death	105	52	38	19	28	6	11	10	100	219	33	73	20	147	271
PF00560	LRR_1	239	180	150	107	413	101	94	71	297	868	230	71	161	195	128
PF00047	ig	173	160	144	156	142	107	133	106	145	278	338	75	157	328	187

Table S5. List of biomineralization-related genes tandemly arranged in the genome. See also Figure 5.

scaffold	annotation	localization of the protein	gene model ID (ver. 2.0)	synonym in gene model ver. 1.0	BLASTP best hit against UniProtKB or NBCI nr description	Accession	E-value
214	nacrein	nacre and prism[a]	pfu_aug2.0_214.1_13802	pfu_aug1.0_33972.1_19513	Nacrein [*Pinctada fucata*]	Q27908	0
	nacrein-like	-	pfu_aug2.0_214.1_13803	pfu_aug1.0_541.1_22209	N66 matrix protein [*P. maxima*]	Q9NL38	3.00E-69
2205	MSI60	nacre[b]	pfu_aug2.0_2205.1_18728	pfu_aug1.1_172742.1_80007	insoluble protein (MSI60) [*P. fucata*]*	BAA20466	0
	MSI60-related	-	pfu_aug2.0_2205.1_18729	pfu_aug1.0_5926.1_52657	MSI60-related protein [*P. fucata*]*	BAL42250	0
932	N19-A	nacre[c]	pfu_aug2.0_932.1_08017	pfu_aug1.0_16924.1_69026	nacre protein (N19) [*P. fucata*]*	BAK57308	3.00E-135
	N19-B4		pfu_aug2.0_932.1_08018	pfu_aug1.0_2495.1_22993	nacre protein (N19) [*P. fucata*]*	BAK57311	4.00E-137
	N19-B5		pfu_aug2.0_932.1_08019	pfu_aug1.0_172333.1_06741	nacre protein (N19) [*P. fucata*]*	BAM77463	4.00E-98
1101	Kunitz/BPTI SPI	nacre[d]	pfu_aug2.0_1101.1_04821	pfu_aug1.0_24634.1_54990	Carboxypeptidase inhibitor SmCI [*Crassostrea gigas*]	K1PX42	6.00E-47
	Kunitz/BPTI SPI		pfu_aug2.0_1101.1_04822	pfu_aug1.0_5601.1_16716	BPTI/Kunitz domain-containing protein 2 [*P. maxima*]	P86963	3.00E-32
	Kunitz/BPTI SPI		pfu_aug2.0_1101.1_04823	pfu_aug1.0_12892.1_39408	BPTI/Kunitz domain-containing protein 5 [*P. margaritifera*]	H2A0P0	1.00E-82
	Kunitz/BPTI SPI		pfu_aug2.0_1101.1_04824	pfu_aug1.0_12892.1_39409	Papilin [*C. gigas*]	K1Q4R7	2.00E-36
	Kunitz/BPTI SPI		pfu_aug2.0_1101.1_04825	pfu_aug1.0_12892.1_39410	BPTI/Kunitz domain-containing protein 3 [*P. margaritifera*]	H2A0N5	3.00E-107
2907	Kunitz/BPTI SPI	nacre[d]	pfu_aug2.0_2907.1_25577	pfu_aug1.0_876.1_36713	BPTI/Kunitz domain-containing protein 4 [*P. margaritifera*]	H2A0N9	2.00E-49
	Kunitz/BPTI SPI		pfu_aug2.0_2907.1_25578	pfu_aug1.0_876.1_36713	BPTI/Kunitz domain-containing protein 4 [*P. margaritifera*]	H2A0N9	1.00E-37
61	shematrin-3	(nacre and prism[e])	pfu_aug2.0_663.1_11057	pfu_aug1.0_312495.1_50334	Shematrin-3 [*P. fucata*]*	BAE93435	1.00E-49
	shematrin-1		pfu_aug2.0_663.1_11058	pfu_aug1.0_24266.1_33421	Shematrin-1 [*P. fucata*]*	BAE93433	0
	shematrin-2		pfu_aug2.0_663.1_11059	pfu_aug1.0_89285.1_56763	Shematrin-2 [*P. fucata*]*	BAE93434	0
2802	shematrin-4B	(nacre and prism[e])	pfu_aug2.0_2802.1_08985	pfu_aug1.0_100445.1_49543	Shematrin-4 [*P. fucata*]*	BAE93436	5.00E-40
	shematrin-4A		pfu_aug2.0_2802.1_08986	pfu_aug1.0_16186.1_10903	Shematrin-4 [*P. fucata*]*	BAE93436	0
618	alveolin-like	prism[d]	pfu_aug2.0_618.1_27594	pfu_aug1.0_89.1_57967	Valine-rich protein [*P. maxima*]	P86947	2.00E-73
	MP10		pfu_aug2.0_618.1_27595	pfu_aug1.0_3035.1_59110	Mantle protein [*P. maxima*]	P86948	1.00E-53
6	chitobiase-1	prism[d]	pfu_aug2.0_6.1_20027	pfu_aug1.0_6745.1_67527	Putative beta-hexosaminidase [*P. margaritifera*]	H2A0L6	1.00E-68
	chitobiase-2		pfu_aug2.0_6.1_20028	pfu_aug1.0_6745.1_67528	Putative beta-hexosaminidase [*P. margaritifera*]	H2A0L6	0
194	Clp-1	prism[d]	pfu_aug2.0_194.1_13762	pfu_aug1.0_14887.1_32490	Putative chitinase 1 [*P. margaritifera*]	H2A0L4	0
	Clp-3		pfu_aug2.0_194.1_13763	pfu_aug1.0_10761.1_31980	Putative chitinase [*P. maxima*]	P86955	0
2116	EGF-like2A	prism[d]	pfu_aug2.0_2116.1_21942	pfu_aug1.0_853.1_22356	EGF-like domain containing protein 2 [*P. margaritifera*]	H2A0L3	5.00E-141
	EGF-like2B		pfu_aug2.0_2116.1_21943	pfu_aug1.0_853.1_22358	EGF-like domain-containing protein 2 [*P. maxima*]	P86954	2.00E-88
429	fibronectin-2	prism[d]	pfu_aug2.0_429.1_30750	pfu_aug1.0_13143.1_39453	Fibronectin type III domain-containing protein 2 [*P. margaritifera*]	H2A0L8	5.00E-172
	fibronectin-1A		pfu_aug2.0_429.1_30751	pfu_aug1.0_9430.1_38910	Fibronectin type III domain-containing protein 1 [*P. margaritifera*]	H2A0L7	5.00E-75
	fibronectin-1B		pfu_aug2.0_429.1_30752	pfu_aug1.0_6844.1_09446	Fibronectin type III domain-containing protein 1 [*P. margaritifera*]	H2A0L7	0
465	peroxidase-like-1	prism[d]	pfu_aug2.0_465.1_17456	pfu_aug1.0_6329.1_09321	Peroxidase-like protein [*P. margaritifera*]	H2A0M7	0
	peroxidase-like-2		pfu_aug2.0_465.1_17457	pfu_aug1.0_32945.1_48096	Peroxidase-like protein [*P. margaritifera*]	H2A0M7	1.00E-126
	peroxidase-like-3		pfu_aug2.0_465.1_17458	pfu_aug1.0_32945.1_48096	Peroxidase-like protein [*P. margaritifera*]	H2A0M7	0
	peroxidase-like-4		pfu_aug2.0_465.1_17459	pfu_aug1.0_33687.1_48123	Peroxidase-like protein [*P. margaritifera*]	H2A0M7	0
	peroxidase-like-5		pfu_aug2.0_465.1_17461	pfu_aug1.0_1885.1_51458	Peroxidase-like protein [*P. margaritifera*]	H2A0M7	5.00E-163
	peroxidase-like-6		pfu_aug2.0_465.1_17462	pfu_aug1.0_11935.1_03146	Peroxidase-like protein [*P. margaritifera*]	H2A0M7	0
242	tyrosinase1A	prism[d]	pfu_aug2.0_242.1_07222	pfu_aug1.0_10251.1_39018	Tyrosinase-like protein 1 [*P. margaritifera*]	H2A0L0	8.00E-169
	tyrosinase1B		pfu_aug2.0_242.1_07224	pfu_aug1.0_10251.1_39018	Tyrosinase-like protein 1 [*P. margaritifera*]	H2A0L0	1.00E-165

[a] Miyamoto *et al*. (1996)[59]
[b] Sudo *et al*. (1997)[60]
[c] Yano *et al*. (2007)[58]
[d] Marie *et al*. (2012)[10]
[e] Localization is inferred based on the gene expression analysis of Yano *et al*. (2006)[57]
* BLASTP best hit against NCBI non-redundant database

Table S6. Hox and neighboring gene models tandemly arranged in the genome. See also Figure 6a.

scaffold ID	gene annotation	gene model ID (ver 2.0)	synonym in gene model ver 1.0	BLAST best hit against UniProtKB			BLAST best hit against *L. gigantea* gene model			BLAST best hit against *C. gigas* gene model		
				description	Accession	E-value	scaffold ID	protein ID	E-value	scaffold ID	protein ID	E-value
	Hox1	pfu_aug2.0_73.1_10231	pfu_aug1.0_2046.1_29956	Homeobox protein Hox-A1[*Heterodontus francisci*]	Q9IA19	6.00E-24		100648	2.56E-23		CGI_10024084	1.00E-64
	Hox2	pfu_aug2.0_73.1_10230	pfu_aug1.1_7850.1_80176	Homeobox protein Hox-A2 [*Bos taurus*]	Q0VCS4	1.00E-45		110623	2.01E-29		CGI_10024086	1.00E-92
	Hox3	pfu_aug2.0_73.1_10229	pfu_aug1.1_3384.1_80162	Homeobox protein HOX3 [*Branchiostoma floridae*]	P50901	1.00E-35	12	56601	1.38E-27	801	CGI_10024087	3.30E-90
73	Hox4	pfu_aug2.0_73.1_10225	pfu_aug1.1_103000.1_80163	Homeobox protein Hox-B4a [*Danio rerio*]	P22574	1.00E-49		110972	3.43E-44		CGI_10024091	1.00E-105
	Hox5	pfu_aug2.0_73.1_10224	pfu_aug1.1_9342.1_80178	Homeobox protein Hox-B5b [*Takifugu rubripes*]	Q1KKX0	3.00E-36		86927	5.08E-60		CGI_10024092	2.00E-54
	-	pfu_aug2.0_73.1_10223	pfu_aug1.0_14207.1_54039	-	-	-	-	-	-	1206	CGI_10002107	7.00E-08
	-	pfu_aug2.0_73.1_10222	pfu_aug1.0_7484.1_09580	-	-	-	-	-	-	1359	CGI_10013240	4.00E-09
	Lox5	pfu_aug2.0_126.1_20292	pfu_aug1.1_422.1_80164	Lox5 hox protein [*Capitella teleta*]	B6RFX8	8.00E-44		89598	5.47E-32		CGI_10026565	7.00E-74
	Antp	pfu_aug2.0_126.1_20291	pfu_aug1.0_422.1_43655	Homeobox protein Hox-A7 [*Heterodontus francisci*]	Q9IA25	3.00E-28	12	177860	1.31E-32	145	CGI_10026565	3.00E-25
	Lox4	pfu_aug2.0_126.1_20290	pfu_aug1.1_5652.1_80179	Lox4 hox protein [*Capitella teleta*]	B6RFX9	8.00E-50		185752	4.63E-45		CGI_10026562	1.00E-108
	-	pfu_aug2.0_126.1_20289	pfu_aug1.0_24639.1_40507	-	-	-	-	-	-	37500	CGI_10002382	2.00E-07
	-	pfu_aug2.0_126.1_20288	pfu_aug1.0_1480.1_22604	Enteropeptidase [*Bos taurus*]	P98072	6.00E-36	238	175076	4.23E-38	86	CGI_10028222	2.00E-92
126	-	pfu_aug2.0_126.1_20287	pfu_aug1.0_1480.1_22604	-	-	-	41	122611	1.89E-44	317	CGI_10019362	5.00E-69
	-	pfu_aug2.0_126.1_20286	pfu_aug1.0_144891.1_49906	Mannosyl-oligosaccharide 1,2-alpha-mannosidase IA [*Sus scrofa*]	O02773	4.00E-97	84	222667	3.73E-174	43842	CGI_10013108	1.00E-120
	-	pfu_aug2.0_126.1_20285	-	Insulin-like growth factor I [*Oryctolagus cuniculus*]	Q95222	4.00E-07	-	-	-	-	-	-
	-	pfu_aug2.0_126.1_20284	pfu_aug1.0_653.1_07611	-	-	-	31	161981	9.24E-28	33704	CGI_10001119	4.00E-29
	-	pfu_aug2.0_126.1_20283	pfu_aug1.0_653.1_07610	-	-	-	-	-	-	-	-	-
	-	pfu_aug2.0_126.1_20282	pfu_aug1.0_24603.1_33450	Importin-5 [*Mus musculus*]	Q8BKC5	0	10	184912	0.00E+00	1827	CGI_10013799	0.00E+00
	-	pfu_aug2.0_126.1_20281	-	cAMP-dependent protein kinase catalytic subunit PRKX *[Homo sapiens*]	P51817	3.00E-68	23	116148	1.02E-76	1688	CGI_10009216	5.00E-55
	Lox2	pfu_aug2.0_93.1_10254	pfu_aug1.1_1857.1_80165	Homeobox protein LOX2 [*Crassostrea gigas*]	K1RXD0	8.00E-58		156932	1.65E-24	247	CGI_10018592	7.00E-50
	Post2	pfu_aug2.0_93.1_10256	pfu_aug1.0_3017.1_01301	Homeobox protein abdominal-B [*Drosophila melanogaster*]	P09087	9.00E-19	12	89720	4.03E-40	1179	CGI_10027388	1.00E-114
	Post1	pfu_aug2.0_93.1_10258	pfu_aug1.0_19935.1_33056	Homeobox protein Hox-C11 [*Homo sapiens*]	O43248	2.00E-17		100031	8.46E-37		CGI_10027385	1.00E-44
93	-	pfu_aug2.0_93.1_10259	pfu_aug1.0_518.1_15112	-	-	-	-	-	-	198	CGI_10027798	3.00E-16
	-	pfu_aug2.0_93.1_10261	pfu_aug1.0_4446.1_52277	-	-	-	-	-	-	22	CGI_10028832	4.00E-08
	-	pfu_aug2.0_93.1_10262	pfu_aug1.0_4446.1_52276	Coatomer subunit zeta-1 [*Bos taurus*]	P35604	4.00E-82	12	156936	7.00E-106	1179	CGI_10027384	4.00E-104
	-	pfu_aug2.0_93.1_10263	pfu_aug1.0_16642.1_61633	SWI/SNF complex subunit SMARCC2 [*Homo sapiens*]	Q8TAQ2	0		177862	0		CGI_10027383	3.00E-06

Shaded scaffolds contain Hox genes.

Table S7. ParaHox and neighboring gene models tandemly arranged in the genome. See also Figure 6b.

scaffold ID	gene annotation	gene model ID (ver. 2.0)	synonym in gene model ver 1.0	BLAST best hit against UniProtKB			BLAST best hit against *L. gigantea* gene model			BLAST best hit against *C. gigas* gene model		
				description	Accession	E-value	scaffold ID	protein ID	E-value	scaffold ID	protein ID	E-value
446	-	pfu_aug2.0_446.1_20790	pfu_aug1.0_26346.1_62427	Armadillo repeat-containing protein 3 [*Homo sapiens*]	Q5W041	5.00E-160	13	111572	2.00E-142	1901	CGI_10015552	0
	-	pfu_aug2.0_446.1_20789	pfu_aug1.0_1405.1_37001	Glutamate receptor ionotropic, kainate 3 [*Macaca fascicularis*]	Q38PU2	3.00E-08	36	233226	7.00E-11		CGI_10015551	8.00E-38
	-	pfu_aug2.0_446.1_20788	pfu_aug1.0_18127.1_03895	Glutamate receptor ionotropic, kainate 3 [*Homo sapiens*]	Q13003	5.00E-66	13	111532	6.00E-106		CGI_10015550	0
	-	pfu_aug2.0_446.1_20787	pfu_aug1.0_12412.1_46500	Glutamate receptor ionotropic, kainate 2 [*Homo sapiens*]	Q13002	8.00E-78		111488	0		CGI_10015549	0
	Gsx	pfu_aug2.0_446.1_20786	pfu_aug1.0_72505.1_41946	GS homeobox 1 [*Homo sapiens*]	Q9H4S2	8.00E-28	80	99857	1.99E-30		CGI_10015548	4.00E-62
	-	pfu_aug2.0_446.1_20785	pfu_aug1.0_17408.1_39942	-	-	-	12	159233	1.04E-34		CGI_10015546	3.00E-62
	Xlox	pfu_aug2.0_446.1_20784	pfu_aug1.0_17408.1_39943	ParaHox homeotic protein xLox [*Sepia officinalis*]	A0A023UHY1	4.00E-48	80	100678	1.70E-38		CGI_10015546	1.00E-52
	-	pfu_aug2.0_446.1_20783	pfu_aug1.0_342779.1_28872	-	-	-		236400	1.52E-10	-	-	-
	Cdx	pfu_aug2.0_446.1_20781	pfu_aug1.0_6325.1_59995	Homeotic protein caudal [*Drosophila melanogaster*]	P09085	1.00E-35	85	197335	3.70E-42	348	CGI_10023003	1.00E-113

Shaded scaffolds contain ParaHox genes.

Table S8. Wnt and adjacent gene models tandemly arranged in the genome. See also Figure 6c.

scaffold ID	gene annotation	gene model ID (ver. 2.0)	synonym in gene model ver 1.0	BLASTP best hit against UniProtKB			BLASTP best hit against *L. gigantea* gene model			BLASTP best hit against *C. gigas* gene model		
				description	Accession	E-value	scaffold ID	protein ID	E-value	scaffold ID	protein ID	E-value
277	wnt9	pfu_aug2.0_277.1_23797	pfu_aug1.0_189.1_36368	Protein Wnt-9a [*Gallus gallus*]	O42280	3.00E-110	107	180028	7.83E-145	825	CGI_10012717	7.00E-66
	wnt1	pfu_aug2.0_277.1_23796	pfu_aug1.0_11928.1_60961	Protein Wnt-1 [*Bombyx mori*]	P49340	1.00E-33		170942	7.00E-86	747	CGI_10017080	3.00E-50
	wnt6	pfu_aug2.0_277.1_23794	pfu_aug1.0_39331.1_62990	Protein Wnt-6 *[Homo sapiens*]	Q9Y6F9	4.00E-73		136505	2.30E-133		CGI_10017079	1.00E-116
	wnt10	pfu_aug2.0_277.1_23793	pfu_aug1.0_16143.1_54282	Protein Wnt [*Crassostrea gigas*]	K1Q987	1.00E-139		136550	3.70E-64		CGI_10017078	1.00E-115
