# Supplementary material for: Bivalve-specific gene expansion in the pearl oyster genome: implications of adaptation to a sessile lifestyle
Source: Zoological Lett. 2016 Feb 18;2:3. doi: 10.1186/s40851-016-0039-2 (PMC4759782; doi:10.1186/s40851-016-0039-2)
Supplement: Additional file 2: — Figure S1. Histograms of sequence coverage depth of each contig. (a) Contig coverage distribution in the initial assembly. The peak at lower coverage (near 26.5x) indicates redundant contigs caused by the heterozygotic nature of the Pinctada fucata genome. The red line shows the fitted normal distribution, where μ = 26.5 and σ = 3. (b) The histogram of contig coverage depth after redundant contigs were removed. (c) The histogram of scaffold coverage depth. These data indicate that redundant sequences in the genome assembly were effectively removed by a sequence similarity and coverage-based method. Figure S2. The numbers of genes corresponding to grouped gene families presented in Fig. 2a and b in the main text. (a–c) The numbers of genes that were assigned by OrthoMCL DB; (a) Pinctada fucata, (b) Crassostrea gigas, and (c) Lottia gigantea. (d–f) The numbers of genes that were not assigned by OrthoMCL DB, while detected in comparisons among three mollusc genomes; (d) P. fucata, (e) C. gigas, and (f) L. gigantea. Mollusc-specific genes shared by all three molluscs are much less abundant than lineage-specific genes. (PDF 213 kb) [file 40851_2016_39_MOESM2_ESM.pdf]

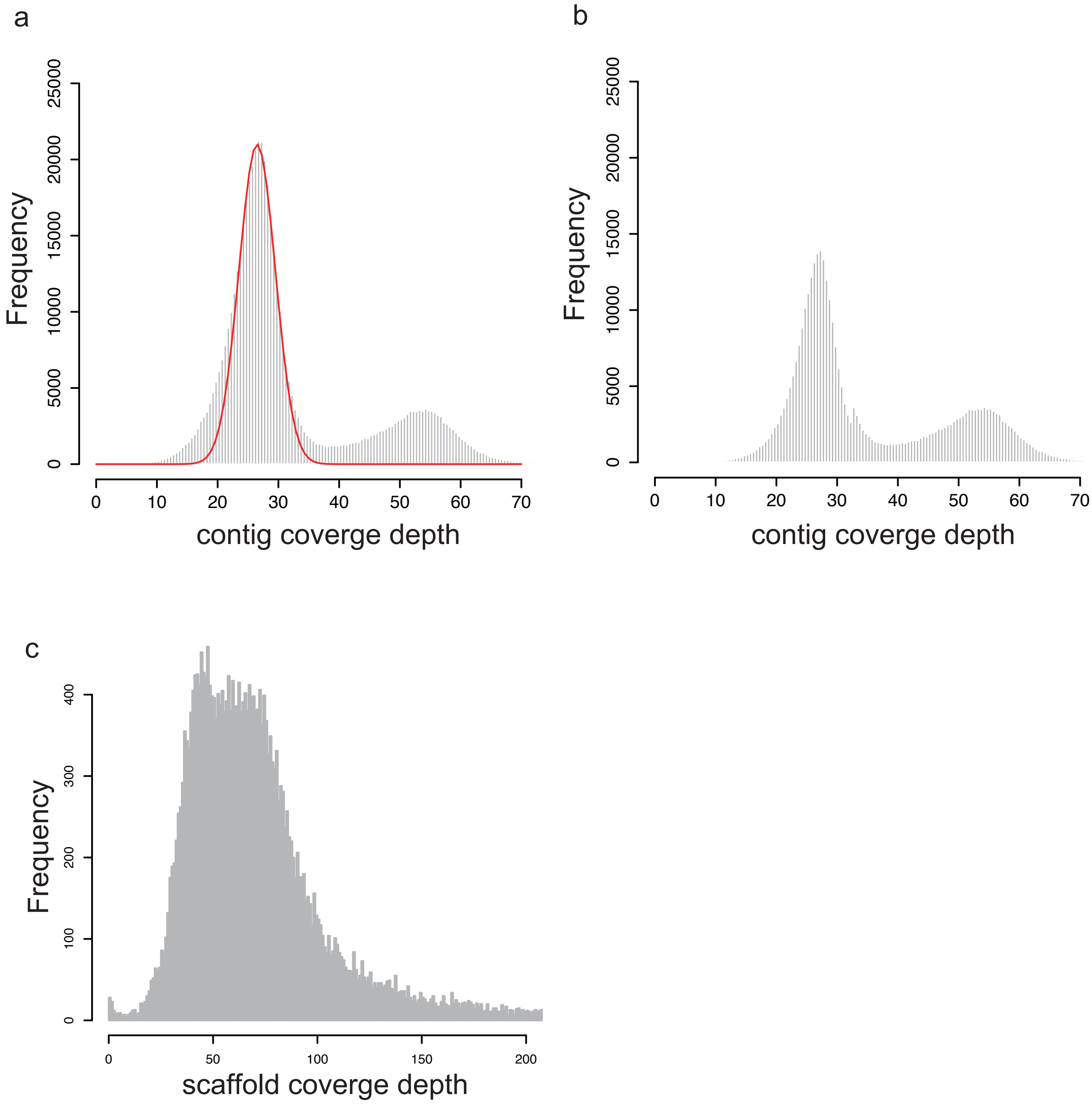

Figure S1. Histograms of sequence coverage depth of each contig. (a) Contig coverage distribution in the initial assembly. The peak at the lower coverage (near 26.5x) indicates redundant contigs caused by the heterozygotic nature of the *Pinctada fucata* genome. The red line shows the fitted normal distribution, where $\mu=26.5$ and $\sigma=3$. (b) The histogram of contig coverage depth after redundant contigs were removed. (c) The histogram of scaffold coverage depth. Those data indicate that redundant sequences in the genome assembly were effectively removed by sequence similarity and coverage based method.

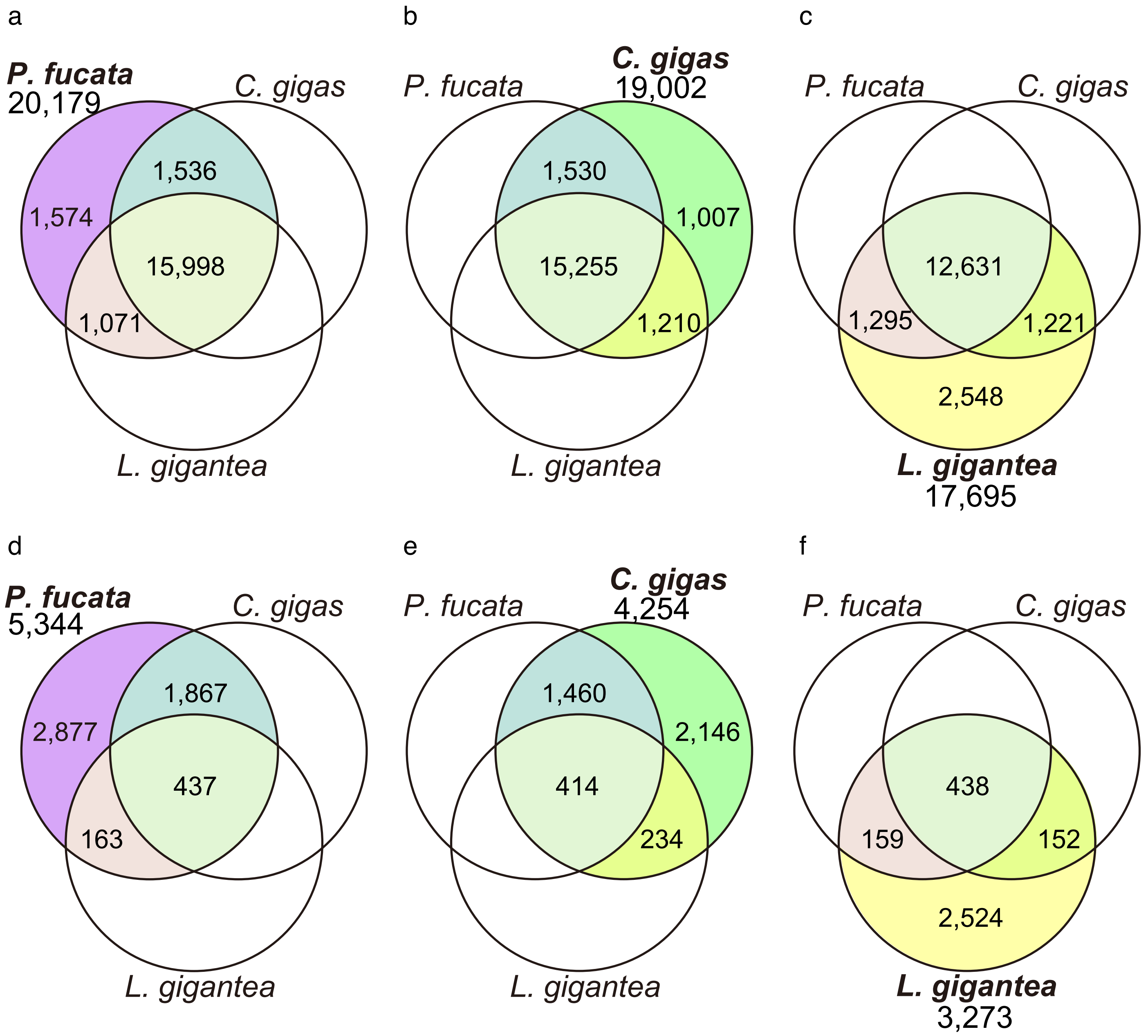

Figure S2. The numbers of genes corresponding to the grouped gene family presented in Figure 2a and 2b in the main text. (a-c) The numbers of genes which are assigned by OrthoMCL DB; (a) *Pinctada fucata*, (b) *Crassostrea gigas*, and (c) *Lottia gigantea*. (d-f) The numbers of genes which are not assigned by OrthoMCL DB, while detected by comparison among three molluscan genomes; (d) *P. fucata*, (e) *C. gigas*, and (f) *L. gigantea*. Mollusca-specific gene families shared by three molluscs are much less abundant than lineage-specific genes.
