# Supplementary material for: Bivalve-specific gene expansion in the pearl oyster genome: implications of adaptation to a sessile lifestyle
Source: Zoological Lett. 2016 Feb 18;2:3. doi: 10.1186/s40851-016-0039-2 (PMC4759782; doi:10.1186/s40851-016-0039-2)
Supplement: Additional file 3: — Assembly strategy. (PDF 97 kb) [file 40851_2016_39_MOESM3_ESM.pdf]

Additional file 3

Assembly strategy

In order to optimize the genome assembly of *Pinctada fucata*, we compared three different assembly strategies. Newbler 2.6 (1), SOAPdenovo2 (2), and Platanus 1.2.1 (3) were tested. After quality trimming, long reads generated by 454 and MiSeq were used for Newbler assembly with default settings, while all reads were processed by SOAPdenovo2 (with default settings except "max_rd_len=250") and Platanus 1. (with default settings except "-μ 0.2"). Consequently, Newbler output the least number of contigs (512,705) and the longest N50 length, compared to the results of SOAPdenovo and Platanus (Additional file 1: Table S2). The total length of contig sequences from SOAPdenovo (1.5Gb) and Platanus (1.8Gb) were considerably longer than the estimated genome size of *P. fucata* (1.1Gb), indicating that these assemblies contain redundant sequences. A subsequent scaffolding process performed with SOAPdenovo and Platanus slightly reduced the total number of sequences (3,971,774 and 4,413,364, respectively), but they were still larger than that of the contig assembly generated by Newbler. Hence, we used Newbler contigs for further processing.

In the primary assembly, large numbers of contigs exhibited lower coverage depth (~26.5), despite ~53-fold sequence data used for the contig assembly (Additional file 2: Figure S1a). The distribution of low coverage depth fits a Gaussian distribution, with $\mu = 26.5$ and $\sigma = 3$. It is presumed that the contigs with less than $26.5 + 3 \times 2 = 32.5$ coverage depth most likely include redundant haplotype copies. In order to filter out the redundancy in the primary assembly, contigs were BLASTN searched against themselves. If a contig fit all the following criteria, we removed it as redundant: i) a contig was shorter than the BLAST best-hit counterpart; ii) the 50% sequence range of the shorter contig was aligned to another contig with sequence identity >85%; iii) sequence coverage depth was 32.5 or lower.

After filtering, 29.5% ((987Mb - 696Mb) / 987Mb) of the total contig assembly was discarded (Additional file2: Figure S1b). Takeuchi *et al*. (2012) showed that 65.3% of the contig genome assembly version 1.0 represents highly heterozygotic regions. In other words, a half of the duplicated contigs (32.6%) should be filtered out from the assembly to obtain a non-redundant, haploid assembly. Therefore, we conclude that an appropriate number of redundant contigs was removed with the method employed here. Furthermore, longer scaffold assembly resulted from subsequent scaffolding using the filtered contig assembly (Additional

file 1: Table S2).

Lastly, we checked sequence coverage depth of the final genome scaffold. illumina paired-end reads were mapped onto the scaffold using Bowtie2 (4) with an option "-N 1", and coverage depth of the scaffold was estimated with SAMtools 1.2 (5). The sequence coverage depth distribution of the scaffolds shows that the peak at lower coverage depth is greatly reduced (Additional file 2: Figure S1c), indicating that the scaffolds represent less redundant, haplotype genome sequences.

References

1. Margulies M, Egholm M, Altman WE, Attiya S, Bader JS, Bemben LA, Berka J, Braverman MS, Chen Y-J, Chen Z *et al*: **Genome sequencing in microfabricated high-density picolitre reactors**. *Nature* 2005, **437**(7057):376-380.
2. Luo R, Liu B, Xie Y, Li Z, Huang W, Yuan J, He G, Chen Y, Pan Q, Liu Y *et al*: **SOAPdenovo2: an empirically improved memory-efficient short-read de novo assembler**. *GigaScience* 2012, **1**(1):18.
3. Kajitani R, Toshimoto K, Noguchi H, Toyoda A, Ogura Y, Okuno M, Yabana M, Harada M, Nagayasu E, Maruyama H *et al*: **Efficient de novo assembly of highly heterozygous genomes from whole-genome shotgun short reads**. *Genome Res* 2014, **24**(8):1384-1395.
4. Langmead B, Salzberg SL: **Fast gapped-read alignment with Bowtie 2**. *Nat Meth* 2012, **9**(4):357-359.
5. Li H, Handsaker B, Wysoker A, Fennell T, Ruan J, Homer N, Marth G, Abecasis G, Durbin R, Subgroup GPDP: **The Sequence Alignment/Map format and SAMtools**. *Bioinformatics* 2009, **25**(16):2078-2079.
